# The hydromechanics in arteriogenesis

**DOI:** 10.1002/agm2.12101

**Published:** 2020-03-16

**Authors:** Tianqi Ma, Yong‐Ping Bai

**Affiliations:** ^1^ Department of Geriatric Medicine Xiangya Hospital Central South University Changsha China

**Keywords:** arteriogenesis, collateral circulation, fluid shear stress, hydromechanics

## Abstract

Coronary heart diseases are tightly associated with aging. Although current revascularization therapies, such as percutaneous coronary interventions (PCI) and coronary artery bypass graft (CABG), improve the clinical outcomes of patients with coronary diseases, their application and therapeutic effects are limited in elderly patients. Thus, developing novel therapeutic strategies, like prompting collateral development or the process of arteriogenesis, is necessary for the treatment of the elderly with coronary diseases. Arteriogenesis (ie, the vascular remodeling from pre‐existent arterioles to collateral conductance networks) functions as an essential compensation for tissue hypoperfusion caused by artery occlusion or stenosis, and its mechanisms remain to be elucidated. In this review, we will summarize the roles of the major hydromechanical components in laminar conditions in arteriogenesis, and discuss the potential effects of disturbed flow components in non‐laminar conditions.

## 1 INTRODUCTION

Increasing age has been identified as an independent risk factor for the incidence of coronary atherosclerosis. According to an age‐period‐cohort analysis of acute myocardial infarction (AMI) in China from 1987 to 2014, population aging has contributed to a steep elevation in mortality due to AMI since 2004.^1^ In terms of elderly patients (more prone to diffused coronary stenosis or multivessel disease), the maximally beneficial results of current strategies, including interventional or surgical revascularization (percutaneous coronary interventions [PCI] and coronary artery bypass graft [CABG]), are limited. Even though PCI improved the clinical outcomes in acute coronary syndromes (ACS) significantly, it was related to a higher incidence of myocardial infarction in stable multivessel coronary disease.^2^ And the application of coronary artery bypass graft surgery in the elderly is restrained by its strict indications, surgical stress, perioperative complications, and needs for specific caregiving programs.^3^ Thus, promoting the formation of coronary collateral circulation through medicine or physical therapy to compensate for myocardial hypoperfusion might be a novel therapeutic strategy for coronary heart diseases in elderly patients.

Arteriogenesis, the primary process of collateral development, has been found to play an essential role in the compensation of ischemic vascular diseases, including myocardial ischemia, stroke, and other peripheral vascular diseases.^4^ Taking myocardial infarction, which is usually caused by coronary atherosclerosis, as an example: because the anatomical features of coronary vessels with their interconnections among arteries form an anastomotic network, it is possible to alleviate the clinical symptoms of myocardial ischemia by developing compensating arteries from pre‐existent anastomotic arterioles.^5^ According to clinical research, associations have been observed between the formation of collateral circulation and the severity of coronary ischemic events.^5^ However, in addition to its heterogeneity among patients, the process of arteriogenesis also presents inherent deficiency in that natural collateral vessels after arterial occlusion only restore 35%‐40% of the maximal conductance normally,^6^ and this does not surpass 50% even in conditions with cytokines or growth factor management.^7^ Thus, it is doubtlessly necessary to understand the triggers, molecular mechanisms, and maintaining factors in arteriogenesis, and develop novel therapeutic strategies for ischemic diseases by prompting collateral development. In this review, mechanical forces in laminar or non‐laminar conditions and their roles in the process of arteriogenesis will be discussed, respectively, to elucidate how alterations of hydromechanics after arterial stenosis or occlusion affect the process of arteriogenesis.

### 1.1 Arteriogenesis triggered by mechanical forces rather than ischemia

Arteriogenesis, the process of vascular remodeling from pre‐existent arterioles to conductance arteries after arterial occlusion, seems to be related to ischemia due to tissue hypoperfusion. However, studies in animal ischemia models have revealed that collateral circulation also occurs in oxygen‐rich sites distant from the hypoperfusion territories,^8^ and an inverse association between the intensity of ischemia and collateral developing speed exists.^9^ Considering the absence of upregulation of hypoxia‐sensitive hypoxia‐inducible factor 1 (HIF‐1α) in collateral regions harvested from rabbits with ischemic hind limb,^10^ the triggering factor of arteriogenesis is not ischemia that stimulates angiogenesis and development of novel vessels from pre‐existent plexus. Instead, the initiation of collateral development is physical‐forces‐dependent.^11^

In the process of arteriogenesis, there is a phenomenon of “pruning,” in which numerous small vessels regress, while several larger arteries remain and grow,^12^ and an artery with hemodynamic stenosis tends to be replaced by a few smaller collateral arteries, rather than by a single conductance vessel.^13^ In terms of morphology, collateral networks are inherently tortuous, with multiple curvatures and non‐physiological angles at their confluences with distal stems. In this way, the natural insufficiency of collateral circulation to compensate for the occlusion‐mediated deficiency in blood flow is reasonable, because additional energy will be lost due to increased resistance in the tortuous collateral circulation with several arteries.^14^

Generally, arteriogenesis happens in response to an arterial occlusion‐mediated sharp elevation in pressure gradients.^15^ As a hemodynamically arterial occlusion occurs, the abrupt interruption of blood flow increases the pressure gradient between proximal and distal arteries with occlusion. Driven by the great ΔP, blood flow tends to take the path of anastomoses with smallest resistance, leading to significant increase in flow passing pre‐existent arterioles and changes in mechanical forces exerted on vascular walls, which will trigger the whole arteriogenesis process by reprogramming endothelial gene expression for NO production, chemokines (monocyte chemotactic protein 1 [MCP‐1]) secretion, adhesion molecules upregulation, and other pro‐angiogenic events.^16^ With the chemotactic effect of MCP‐1, circulating monocytes and T‐cells are attracted, adhere, activated, and infiltrate into the endothelium layer or adventitial space of these arterioles.^17^ These monocytes play a pivotal role in collateral development by secreting proteases like matrix metalloproteinases to degrade extracellular matrix,^18^ chemokines like tumor necrosis factor‐α (TNF‐α) to recruit more circulating monocytes to the collateral region, and growth factors that prompt smooth muscle cells (SMCs) proliferation.^19^ Then the medial SMCs proliferate with the switch of their phenotype, actin polymerization, and maturation. After NO‐induced vasodilation and afterward arterial remodeling, the pre‐existent arterioles develop to collateral vessels at conductance levels, which are equipped with the capacity to compensate for occlusion‐mediated hypoperfusion.^20^

## 2 THE HYDROMECHANICS OF ARTERIOGENESIS IN LAMINAR CONDITIONS

**Figure 1:**
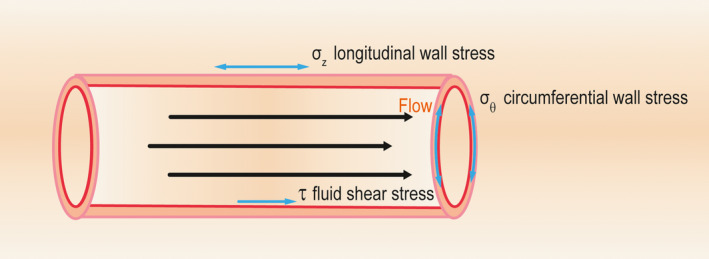
The simplified hemodynamic patterns of collateral arterioles in ideal laminar conditions. Eliminating the disturbing effects of the inherent tortuosity of anastomotic vessels on hydrodynamics, the major hydrodynamic components are the flow‐dependent fluid shear stress (FSS) and pressure‐derived stresses, such as circumferential wall stress (CWS) and longitudinal wall stress (LWS). Among them, the tangential FSS acts on the endothelial layer of the vascular wall, while the perpendicularly exerted CWS and the axial LWS exhibit direct effects on the whole vascular wall

Before the formation of hemodynamically relevant stenosis or occlusion in conductance arteries, there might be only a little blood flow through the preexistent anastomoses, while occlusion‐mediated steep pressure gradient drives substantial blood flow via these collateral arterioles unidirectionally, leading to significant changes in hydromechanics acting on the whole collateral circulation. Assuming the hemodynamic pattern in collateral anastomoses exposed to unidirectional blood flow is laminar, the primary mechanical forces acting on the vascular wall include flow‐dependent fluid shear stress (FSS) and pressure‐dependent stresses, like circumferential wall stress (CWS) and longitudinal wall stress (LWS; Figure 1).

### 2.1 The role of FSS in arteriogenesis

Resulting from the friction of floating blood against the vascular wall, fluid shear stress acts tangentially on the luminal surface of the collateral arterioles, and its direct effects are restrained within the endothelial layer.^21^ Because it is relatively hard to measure FSS directly in collateral arterioles,^22^ several studies have assessed it using measurable blood flow (*Q*) and vascular radius (*r*): $\tau =\frac{4\eta Q}{\pi r^{3}}$ (*τ*: FSS, *η*: blood viscosity). According to the equation, the shear force is flow‐dependent, indicating the flow‐related aggravation of FSS in pre‐existent anastomoses after arterial occlusion.

To date, results obtained from various experimenting animal models and cultured cells in vitro have revealed the pivotal role of shear force in both initial vasodilation and chronic vascular remodeling events of arteriogenesis. According to results obtained from canine carotid arteries with manipulated blood flow,^23^ vascular adaptations to flow alterations were related to altered flow‐oriented force (ie, artery enlargement following the increased flow and vascular wall thickening or artery atrophy in response to decreased blood flow were attempts to restore the altered wall shear stress). In rat intestinal arteries ligation models, initially post‐ligation collateral growth rates and vasodilation depended on different blood flow and shear rates at certain sites, and collateral networks at those regions with the highest blood flow and shear force developed best.^24^ In this way, some researchers developed the “shear stress set point” theory, which means that blood flow or flow‐derived shear stress is maintained to an optimal value by feedback mechanisms.^25^ Cells with higher expression of VEGFR3 tend to have a lower set point and are more sensitive to shear stress, compared with those with lower expression of VEGFR3.^26^ Furthermore, the epigenetic effect, DNA methyltransferase 1 (DNMT1)‐dependent DNA hypermethylation, augments the set point, decreasing endothelial mechanosensitivity, and restricting FSS‐induced arteriogenesis.^27^ In the process of arteriogenesis, alteration of wall shear force (initially abrupt elevation followed by gradual normalization) and FSS‐induced regulation of endothelial growth factors are associated with collateral vascular remodeling events, including hyperplasia of intimal, medial, and adventitial layers at a different time, extracellular matrix reconstruction, and luminal expansion.^28^, ^29^ Even after the normalization of FSS (the shear force should drop ideally with vascular radius in third power as vasodilation occurs), the initially elevated FSS‐stimulated collateral development continues to develop collateral arteries that share identical features with normal arteries.^30^ To elucidate the effects of FSS on arteriogenesis more specifically, an arteriovenous shunt was created in hind limbs of pigs or rabbits to provide persistently elevated fluid shear stress.^31^ The prolonged aggravation of wall shear force led to normalization of maximal conductance and significant development of collateral circulation by prompting cellular proliferation, monocytes adhesion, and cytoskeleton reorganization, directly proving the pivotal role of FSS in stimulating arteriogenesis.

Given the triggering role that FSS plays in arteriogenesis, how does the mechanical alteration (ie, abruptly elevated wall shear stress) affect endothelial cells (ECs) and evoke subsequent biochemical cascades involving the whole vascular wall? The mechanosensation and signal transduction here have not been elucidated well, involving multiple ion channels, G‐proteins, endothelial cytoskeleton, endothelial surface glycocalyx, and other complexes as mechanoreceptors. Initially, the opening of chloride channels, like FSS‐sensitive endothelial volume‐activated outwardly rectifying Cl^‐^ channels (VRAC)^32^ in response to EC swelling was regarded as the first step of endothelial mechanosensation.^33^ However, as the establishment of Ingber’s “tensegrity” model,^34^, ^35^ in which a tensegrity structure formed by cytoskeleton coupling with cell membrane serves as a sensor, the mechanosensing role of the whole endothelial cell has been widely discussed.^36^ When wall shear force is exerted on ECs apically, the integrated, dispersive network of cytoskeletal polymers transfer the mechanical information to sites with membrane attachment where allosteric changes occur in downstream proteins.^37^ Also, Osborn et al^38^ depicted the depolymerization of net cytoskeleton as the earliest phase of endothelial responses to shear stress. Endothelial surface glycocalyx, functioning as a mechanoreceptor and transducer on the luminal surface of endothelial cells, has been identified as necessary to the shear stress‐induced response of the endothelial cytoskeleton and endothelial nitric oxide synthase (eNOS) activation.^39^ The components of the glycocalyx, syndecans linked with the cytoskeleton, have been suggested to play a crucial role in the translation process from mechanical stimuli to biochemical events by participating in the interaction with cytokines, cooperating with integrins, and prompting flow‐related NF‐κB activation,^40^, ^41^, ^42^ particularly the involvement of syndecan‐1 in the phosphoinositide 3‐kinase/Akt/endothelial nitric oxide synthase (PI3K/Akt/eNOS) pathway.^43^

Besides, cation channels, like mechanosensitive calcium‐channel TRPV4 (Ca^2+^‐channel transient receptor potential cation channel, subfamily V, member 4), and certain mechanosensory complexes, like platelet endothelial cell adhesion molecule‐1 (PECAM‐1), vascular epithelium‐cadherin (VE‐cadherin), and vascular endothelial growth factor receptor 2 (VEGFR2), also exhibit specific functions as mechanoreceptors and transducers in shear stress‐induced arteriogenesis.^44^, ^45^ And the heterotrimeric G‐proteins, in combination with seven transmembrane receptors, also participate in fluid shear stress‐initiated signaling cascades via interactions with other mechanosensors.^46^ For instance, the Gq/G11 protein, which forms a heparan sulfates‐stabilized complex with PECAM‐1,^47^ mediates the activation of the endothelial mechanosensory complex consisting of PECAM‐1, VE‐cadherin, VEGRR2, and PECAM‐1. In this complex, the direct transducer of shear stress is vital for Src family kinase action, which is required for shear stress‐induced and PI3K‐dependent activation of integrin. In the whole process, the Src‐dependent activation of VEGFR2 is essential for PI3K activation, while VE‐cadherin acts as an adaptor, regulating the formation of PI3K activation‐related complex.^45^ Besides, the increase in the intracellular calcium concentration related to majorly cation channels has been revealed as one of the earliest events after shear stress stimuli, and the activation of chloride channels, such as VRAC, can lead to a Cl^‐^ influx, increasing the driving force for Ca^2+^ influx additionally.^33^ For instance, the mechanosensitive calcium channels TRPV4 are upregulated in the collateral vessels of pig hindlimb models with A‐V shunt. This leads to a sustained calcium influx to cytoplasm and nucleus, which prompts the activation and translocation of certain calcium‐sensitive transcriptional modulators, such as nuclear factor of activated T‐cells, cytoplasmic, calcineurin‐dependent 1 (NFATc1), CaN, and myocyte enhancer factor 2C (MEF2), accompanied by the inhibition of the transcriptional inhibitor Kv channel interacting protein 3, calsenilin (CSEN).^44^ Among these transcriptional regulators, the deactivation of repressor CSEN might activate AP‐1‐dependent transcriptional regulation in collateral development as in pain modulation, since AP‐1‐associated signal cascades are also crucial in arteriogenesis.^48^ Besides, there are also other mechanosensitive receptors or cation channels that might act as mechanoreceptors in shear stress‐induced collateral development. The mechanosensitive cationic channel Piezo1 in endothelial and vascular smooth cells can be activated by chemicals or mechanical stimuli, such as blood‐flow‐derived shear force and elevated blood pressure‐related stretch in the cell membrane, and initiate cationic, inward currents of Na^+^, K^+^, and Ca^2+^.^49^, ^50^ In the regulation of vascular tone and blood pressure, shear stress‐activated Piezo1 contributes to eNOS phosphorylation and NO generation via Gs‐coupled mechanosensory complex (PECAM‐1, VE‐cadherin, and VEGFR2)‐mediated PI3K/Akt pathway.^51^, ^52^ In angiogenesis, Piezo1‐related calcium influx plays a vital role by activating matrix metalloproteinase‐2 and membrane type 1 matrix metalloproteinase.^49^ Piezo1 is also required for the blood flow‐induced alignment of EC and the determination of vascular tube length and branch points.^50^, ^53^ Thus, it is rational to speculate the association between the mechanosensory Piezo1 and shear stress‐induced arteriogenesis.

In FSS‐related mechanotransduction in arteriogenesis, there are also other molecules involved. Endothelial Shc acts as an adaptor of endothelial responses to FSS and mediates the activation of the NF‐κ‐light‐chain‐enhancer of activated B‐cell‐mediated inflammatory pathway and Notch pathway, both of which are necessary to inflammation and vascular remodeling.^54^ Moreover, in response to shear stress, endothelial cells were observed to release extracellular RNA (eRNA), which induces VEGFR2‐dependent release of von Willebrand factor (vWF) from platelets and therefore triggers the inflammatory process in arteriogenesis.^55^ The energy and redox sensor, AMP‐activated protein kinase α1 (AMPKα1), prompts collateral formation by enhancing NF‐κB‐dependent generation of growth factors, such as transforming growth factor‐β (TGF‐β), when activated by fluid shear stress.^56^ Following the signal transduction from alterations in FSS to biochemical signaling cascades discussed, the modulation of endothelial shear stress‐responsive elements that exist in the prompters of certain genes, including NOS, platelet‐derived growth factor, vascular cell adhesion molecule‐1 (VCAM‐1) and MCP‐1, and related transcriptional factors, like nuclear factor kappa‐B (NF‐κB), early growth response gene 1 (Egr‐1), and activator protein 1 (AP‐1), lead to a series of arteriogenic events.^20^ The molecular mechanisms in the whole process of FSS‐induced collateral development have been reviewed elsewhere.^57^, ^58^

Given the pivotal role that FSS plays in arteriogenesis, is it the single trigger of collateral circulation? There are still a few questions to answer. First, wall shear force is relatively weaker in comparison with pressure‐generated forces, which is two orders of magnitude higher.^20^ And according to the formula $\tau =\frac{4\eta Q}{\pi r^{3}}$ (*τ*: FSS, *η*: blood viscosity), FSS should drop ideally with vascular diameter in third power as arteriogenesis proceeds, which is consistent with the premature normalization of initially elevated FSS before the complement of collateral development in mesenteric arteries ligation models.^28^, ^59^ The FSS is so weak that its direct implications are limited within the vascular endothelium layer, while the whole process of arteriogenesis involves remodeling of the intima, media, adventitia layers, and degradation of the extracellular matrix. Given the fact that there have been no junctions passing paracrine signals between ECs and SMCs observed in collateral vessels,^20^ besides the diffusible vasodilators like NO, are there any other intercellular structures or chemicals that facilitate the signal transduction from endothelial cells to other layers beyond internal elastic lamina? In rat intestinal arteries ligation models, the progress of arteriogenesis after prenormalization of FSS^59^ sheds light on the existence of FSS‐related signaling cascades or other factors, like circumferential wall stress, that maintain collateral development. Besides, exposure of cultured endothelial cells to shear stress downregulated MCP‐1 expression and inhibited monocytes adhesion,^60^ and the supernatants from FSS‐stimulated ECs did not prompt MCP‐1 expression in SMCs,^48^ which were a contrast to results obtained in vivo. Thus, the implications of other factors, including pressure‐derived forces and biochemical signals on arteriogenesis, cannot be eliminated simply.

### 2.2 The role of CWS in arteriogenesis

Circumferential wall stress (CWS), mainly derived from intraluminal blood pressure, is exerted perpendicularly against the vascular wall. According to the equation $\sigma_{\theta }=\frac{\mathrm{pr}}{h}$ (*σ_θ_*: CWS, *p*: pressure on the vascular wall, *r*: vascular radius, *h*: height of the vascular wall), it will be augmented by elevations in blood pressure and vascular diameter, or vascular wall thinning without matrix digestion.

In 1967, Schaper^61^ observed a considerable increase in CWS in early collateral development and attached this force to more importance on the basis that it is two orders of magnitude larger than flow‐derived forces. Even though afterward FSS is widely recognized as the major triggering force in collateral development, the premature normalization of initially elevated FSS due to vasodilation sheds light on the existence of CWS‐generated effects.^28^ The exposure to cyclic stretch upregulates MCP‐1 in human ECs and SMCs significantly, and in isolated second branches of mouse mesenteric arteries with proarteriogenic perfusion mimicking post‐occlusion, hemodynamic conditions in anastomoses (calculated control condition: FSS ≈ 2.17 Pa, CWS ≈ 6480 Pa; calculated proarteriogenic condition: FSS ≈ 2.66 Pa; CWS ≈ 14 665 Pa), the removal of FSS‐sensitive endothelial layers did not affect MCP‐1 expression in media layers, indicating the role CWS plays in arteriogenesis.^48^ However, during the collateral development of rat mesenteric arteries, the relationship between medial thickness and luminal radius kept constant, maintaining vascular CWS,^30^ which was estimated to deviate within 7%.^28^ Thus, the role of CWS in arteriogenesis is still questionable.^20^

According to a theoretical model simulating hemodynamic and metabolic alterations‐stimulated vascular remodeling responses, the tight association between FSS and CWS dynamics exists,^62^ and maintaining the relationship between the two forces is regarded as design principals of collateral circulation.^63^ In this way, the initial FSS‐induced vasodilation might elevate CWS via thinning of the pressure‐bearing vessel wall, which stimulates SMCs proliferation as negative feedback of CWS regulation. In this way, the process of arteriogenesis is triggered and maintained.

### 2.3 The role of LWS in arteriogenesis

Longitudinal wall stress (LWS) or axial stress with scarce data in vivo due to the difficulty of measurements in vivo serves as a modulator of vascular length adaptation. When vessels were subjected to longitudinal stretch, or relative stretch occurred as vascular diameter or wall thickness decreases, the elevated LWS initiated a series of vascular remodeling events involving medial SMCs and matrix as adaptations,^64^ while decreased LWS did not modulate vascular length to be shortened. In collateral networks after arterial occlusion, the vascular tortuosity suggested profound vascular growth and decreased axial tension, which was not normalized inherently.^64^

## 3 THE HYDROMECHANICS OF ARTERIOGENESIS IN NON‐LAMINAR CONDITIONS

Given the inherent tortuosity and irregularity of collateral circulation, it is difficult for experiments and discussions over arteriogenesis in laminar conditions to imitate the practical and complex hemodynamics in anastomoses with curvatures, segmental diameter alterations, and non‐physiological angles.^65^ In the initially collateral development of canine coronary vessels, the observed patterns of endothelial surface (streams, whorls, and non‐oriented mosaics) hinted at the existence of non‐laminar flow patterns, such as jets, eddies, and low shear flow.^66^ And due to the size and complex morphology of pre‐existent arterioles and collateral networks, it is relatively hard to analyze the non‐laminar hydromechanics in arteriogenesis in vivo or mimic in vitro. Therefore, the effects of exposure to a single flow component in the non‐laminar environment on incubated vascular endothelium are discussed to study the possible role of the non‐laminar hydromechanics in arteriogenesis.

### 3.1 Pulsatile shear stress

Exercise training elevated pulsatile shear stress exerted on ECs, which prompts eNOS and NO generation in endothelial cells and thus arteriogenesis.^67^ In the co‐culture system of human coronary artery endothelial cells and human coronary artery smooth muscle cells, exposure of human coronary artery endothelial cells to pulsatile shear stress of a “high” level of about 1.24 Pa prompted the arteriogenic placental growth factor expression and secretion from endothelial cells via a signaling pathway involving NADPH oxidase 4 (Nox4) and H_2_O_2_, in which the EC‐SMC interaction was vital.^68^ Increased pulsatile flow in a mouse femoral artery model upregulated TNF‐α, intercellular cell adhesion molecule‐1 (ICAM‐1), TGF‐β, and the transcription factor Egr‐1 significantly, accompanied with increased monocytes adhesion statically.^69^ Besides effects on ECs, pulsatile shear stress also attenuated the enhanced SMCs migration induced by EC‐derived mitogens and prompted SMCs proliferation.^70^

### 3.2 Oscillatory shear stress

Compared with laminar shear stress, which downregulated VCAM‐1, chronic exposure of human endothelial monolayers to oscillatory shear stress prompted the expression of adhesion molecules, such as VCAM‐1, ICAM‐1, E‐selectin, and cytokines, enhancing monocytes adherence, which might involve an oxidative pathway.^71^ Consistently, oscillatory shear stress upregulated heme oxygenase‐1 (HO‐1) in ECs for more than 24 hours without significant implications on superoxide dismutase, while that upregulation induced by steady shear stress was as transient as 5 hours accompanied by a chronic elevation in superoxide dismutase, indicating the pro‐oxidant role this flow component plays.^72^

### 3.3 Turbulent shear stress

With novel tools, such as DNA microarray gene expression analysis and high‐throughput genomic analysis, researchers have found that the effects of laminar FSS or turbulent shear stress (TSS) on endothelial phenotype were distinct from each other, with specific genes being upregulated by laminar FSS and downregulated by TSS, or being upregulated similarly by both stimuli.^73^ With no observed implications on ICAM‐1 expression or cell alignment of endothelial cells, TSS exhibits a proliferative promotion effect on endothelial cells by stimulating DNA synthesis, especially those genes of cell cycle proteins.^74^ Moreover, distinct from the anti‐atherogenic effects of laminar shear stress via downregulation of atherogenic factors like ET‐1 and PAI, TSS regulated expression of a series of genes involved in atherosclerotic vascular remodeling, such as the upregulation of tPA, uPA, and downregulation of their inhibitor gene.^75^

### 3.4 Temporal and spatial gradients

Given the complex hemodynamic environment in collateral circulation with inherent curvatures, single endothelial cell of anastomoses is also exposed to temporal and spatial gradients of shear stress (ie, rapid alteration of shear stress over a short duration at the same location and different levels of shear stress between two close points of a cell simultaneously). The temporal gradients of shear stress stimulate endothelial cell proliferation via activation of extracellular signal‐regulated kinases 1 and 2 (ERK1/2) pathway,^76^ involving the upstream components, including G proteins, reactive oxygen species (ROS), and NO production,^77^ whereas steady shear stress does not exhibit similar effects.^78^ And temporal gradients of shear stress also exhibit enhancive implications on the expression of transcription factor c‐fos and connexin43, which was opposite against that of laminar shear stress.^79^ The effects of wall shear stress gradients on endothelial cell function depend on the signs and relative magnitude of the gradient, that is, positive wall shear stress gradients at high magnitude prompted EC apoptosis, proliferation, and inhibited flow‐mediated EC alignment, and vice versa.^80^, ^81^ In terms of transcription factors, spatially disturbed laminar shear stress promoted the nuclear localization of transcription factors, including NF‐κB, Egr‐1, c‐jun, and c‐Fos, which function to mediate shear stress‐induced endothelial gene regulation by interacting with certain shear stress‐responsive elements in both EC populations and individuals.^82^

Despite the enhancive effects of pulsatile shear stress on arteriogenesis, most implications of non‐laminar hemodynamic components on vascular endothelial cells and smooth muscle cells discussed above were obtained from models mimicking the disturbed laminar flow condition at atherosclerosis‐prone sites like arterial bifurcations, which were designed to study the role of disturbed flow patterns in atherosclerosis. Even though arteriogenesis might happen near those atherosclerosis‐prone sites with hemodynamic stenosis, it does not mean that it is proper to reckon the similar effects of these non‐laminar flow components in collateral development. And given the multiple and distinct effects that the single flow component exhibits on the vascular wall, it is necessary to figure out and simulate the specific hemodynamics in collateral networks in order to study the hydromechanics of arteriogenesis in non‐laminar conditions.

## 4 SUMMARY

As the trends of population aging are predominant, the incidence of coronary heart disease, a disease of aging, has been elevated for a long time, increasing the consumption of public health resources and reducing the quality of life individually. Compared with PCI or CABG, the promotion of coronary collateral circulation via non‐invasive approaches might have potential advantages in the treatment of diffused coronary stenosis or multivessel heart disease in the elderly,^83^ which urges researchers to elucidate the detailed mechanism in the whole process of arteriogenesis. In the sections above, we concentrated on the hydrodynamics in collateral development, summarized the major hemodynamic components and their contribution to arteriogenesis in laminar conditions, and discussed the potential effects of other components in non‐laminar or disturbed flow. In this section, the arteriogenesis in the context of aging and exercise will also be discussed.

Based on the extensive experimental data obtained from various animal artery ligation models, cultured cells in vitro, and mathematical models, it has been extensively acknowledged that pressure gradient‐mediated elevated FSS after acute artery occlusion plays a crucial role in triggering arteriogenesis afterward. Nevertheless, the detailed mechanosensation and signal transductions of FSS‐induced arteriogenesis need to be elucidated, and several doubts, like the paradoxical effects of elevated flow shear stress or NO on endothelium‐derived MCP‐1 and monocytes adhesion in vitro or vivo, still exist. And Sager et al^84^ proposed that collateral blood flow or flow shear stress and NOS expression were initially reduced within 12 hours after acute arterial occlusion and then elevated during arteriogenesis, allowing macrophages to accumulate and thus enhancing collateral development. In addition to elevated FSS, the implications of CWS to stimulate SMCs proliferation and enhance MCP‐1 expression were also observed in the process of arteriogenesis. Besides, the natural tortuosity of collateral vessels hints at the non‐laminar flow patterns existing in practical sites of arteriogenesis with potentially distinct implications on arterial wall components, especially endothelial cells, which should not be eliminated either.

In a particular context of aging, a widely acknowledged risk factor for cardiovascular diseases, the ability to develop collateral circulation in compensation for conductance arteries occlusion is restrained by increasing age,^85^ with fewer post‐occlusion collateral networks.^86^ In addition to the effects of aging on vascular morphology and compliance, the recession of arteriogenesis and flow‐induced vascular remodeling with aging is attributed partly to the malfunction of several mechanosensitive signaling pathways in vascular aging. The impairment of flow‐induced and endothelium‐mediated vasodilation with increasing age has been observed in several studies, featured by a decline in NO generation from endothelium in response to shear stress.^87^ In the system of $O_{2}^{-}{\text{/H}}_{2}O_{2}$ , the balance of which is vital to flow‐induced vasodilation, aging contributes to the disruption of the balance and increased production of hydroxyl radical, leading to impaired NO‐ and H_2_O_2_‐related signaling.^88^ In the coronary arterioles from Fischer‐344 rats, despite the maintenance of Ach‐induced signaling pathway involving G protein‐coupled receptors, aging constrained flow‐induced, NO‐dependent vasodilation by impairing VEGFR2‐mediated PI3‐kinase/Akt pathway partly.^89^ And the shear stress‐sensitive transcription factor, Kruppel‐like factor, which is majorly mediated by flow, has been observed in aging‐related impairment of shear stress sensing and afterward mechanotransduction.^90^, ^91^

Despite the aging‐related impairment of the capacity of arteriogenesis, appropriate exercise or physical activity might be prescribed for peripheral artery disease or chronic coronary diseases, stimulating the development of collateral networks according to clinical trials.^92^ Exercise with distinct forms in mode and intensity leads to distinct patterns of hemodynamic changes and elicits corresponding responses in vascular walls.^93^ In general, physical activity increases heart stroke, arterial pressure, increases both the magnitude and frequency of shear stress,^94^ and elevates circumferential wall stress, and the frequency of pulsatile changes.^95^ In collateral vessels featured with curvatures, the exercise‐triggered pulsatile changes in blood flow, pressure, and vascular diameter tend to be asynchronous other than the synchronous patterns in aorta and contribute to pro‐atherogenic events in vascular walls,^96^ while exercise‐induced significant elevations in FSS and blood flow (which can be four‐ to six‐fold) lead to anti‐atherogenic events and enhance collateral development.^97^

Due to the difficulty of measuring hydromechanical alterations in pre‐existent arterioles after hemodynamic stenosis or obstruction of conductance arteries in animal models, several studies applied theoretical formulas to assess shear stress and pressure‐derived stresses, narrowing the applications of their conclusions within the laminar conditions, which might fail to reflect the actual alterations in hemodynamics directly. Thus, it is necessary to develop new models of extracorporeal circulation for further research of hydromechanics in collateral circulation after stenosis or obstruction of conductance arteries, especially coronary vessels.

## CONFLICTS OF INTEREST

Nothing to disclose.

## AUTHOR CONTRIBUTIONS

Yong‐Ping Bai is responsible for the conception of the review. Tianqi Ma collected, organized, and wrote the manuscript.
